# A Novel Approach to the Management of Children with Primary Nocturnal Enuresis

**DOI:** 10.3390/children12091128

**Published:** 2025-08-27

**Authors:** Buket Esen Agar, Metin Kaya Gurgoze, Aslihan Kara

**Affiliations:** 1 Department of Pediatric Nephrology, Faculty of Medicine, Firat University, Elazig 23200, Turkey; aslihankara@firat.edu.tr; 2 Department of Pediatric Nephrology and Rheumatology, Faculty of Medicine, Firat University, Elazig 23200, Turkey; mkgurgoze1@firat.edu.tr

**Keywords:** child, enuresis, vitamin B12, 25-hydroxyvitamin D

## Abstract

Background/Objectives: Primary nocturnal enuresis (PNE) is a common condition that adversely affects the quality of life of both children and their families. It is known to have a multifactorial pathogenesis. This study aimed to evaluate serum levels of 25-hydroxyvitamin D (25OHD), vitamin B12, folic acid, and ferritin in children diagnosed with PNE and to investigate the impact of correcting detected deficiencies on the number of wet nights. Methods: A total of 150 pediatric patients diagnosed with monosymptomatic primary nocturnal enuresis (PNE) who had previously undergone standard urotherapy without clinical improvement were included in this study. Serum levels of vitamin B12 and 25-hydroxyvitamin D (25OHD) were assessed, and patients with deficiencies were identified. Vitamin supplementation was administered to those with deficient/insufficient levels. The number of wet nights was recorded at monthly follow-up visits to monitor clinical response. Results: Only 14% of the 150 patients had no detectable vitamin deficiencies. A deficiency in serum vitamin B12 levels was observed in 78.6% of patients, while 41.3% had reduced 25-hydroxyvitamin D (25OHD) levels. Concurrent deficiencies in both 25OHD and vitamin B12 were detected in 34% of the patients. No folate deficiency was observed in any patient. Notably, vitamin supplementation alone resulted in successful enuresis management in 77.6% of the patients. Conclusions: A high prevalence of vitamin B12 and 25-hydroxyvitamin D (25OHD) deficiencies was identified among patients diagnosed with primary nocturnal enuresis (PNE). Significant improvements in nocturnal dryness were achieved solely through correction of these deficiencies, without the use of desmopressin therapy. These findings suggest that targeted vitamin supplementation may play a crucial role in enhancing the success rate of standard urotherapy in the management of PNE.

## 1. Introduction

Primary nocturnal enuresis (PNE), or nighttime bedwetting, is one of the most common disorders in childhood [1]. PNE is defined as involuntary urination during sleep in children over the age of five who have no congenital or acquired defects of the central nervous system (CNS) [2]. The reported prevalence of PNE varies between 4% and 24%, depending on the diagnostic criteria used [3].

Multiple factors have been implicated in the pathogenesis of primary nocturnal enuresis (PNE), including nocturnal polyuria, reduced bladder capacity, sleep and circadian rhythm disturbances, and genetic predisposition [1]. Although delayed maturation of the central nervous system (CNS) has also been widely discussed as a contributing factor, the precise mechanisms underlying PNE remain incompletely understood [4,5].

Several studies have demonstrated that vitamin B12 deficiency in children may lead to involuntary urination and defecation, potentially due to demyelination-associated axonal degeneration [6]. The resulting disruption of myelin homeostasis may contribute to neurological impairment in the pediatric nervous system [7].

Vitamin D (25-hydroxyvitamin D; 25OHD) receptors are widely distributed throughout the body, including in both skeletal and smooth muscle cells, as well as in the detrusor muscle and the urothelium of the bladder. Vitamin D is thought to modulate bladder function by suppressing sensory signaling during the bladder filling phase, thereby helping to reduce detrusor overactivity. A deficiency or insufficiency in vitamin D may lead to uncontrolled bladder contractions and contribute to the development of an overactive bladder. Furthermore, low vitamin D levels have been shown to affect renal expression of endothelin-1 and reduce the activity of epithelial sodium channels, leading to increased natriuresis [8,9,10]. Clinical evidence also suggests that vitamin D plays a role in the regulation of sleep patterns [11].

The aim of our study was to report the serum levels of 25OHD, vitamin B12, folic acid, and ferritin in pediatric patients with PNE, a topic with limited existing literature and to evaluate the effect of vitamin B12 and/or vitamin D supplementation on the frequency of enuretic episodes in patients found to have deficiencies.

## 2. Materials and Methods

The study included 150 pediatric patients who were diagnosed with monosymptomatic PEN by a pediatric nephrologist, who had been given standard urotherapy training without any benefit, and who had no history of desmopressin use. The present study was conducted in a prospective design. Nocturnal enuresis was defined as repeated involuntary urination during sleep occurring at least twice per week in children aged 5 years or older. Monosymptomatic PNE was defined as enuresis persisting for more than six months during sleep in the absence of bladder dysfunction or lower urinary tract symptoms [12].

Patients with underlying renal or urinary tract disorders, urinary tract infections, a history of medication use, diabetes mellitus, diabetes insipidus, psychiatric conditions (e.g., autism spectrum disorder, attention-deficit/hyperactivity disorder), structural central nervous system abnormalities (such as spinal cord disorders, seizures, or head trauma) were excluded from the study.

For each participant included in the study, body weight, height, blood pressure, urinary symptoms, history of urinary tract infections and constipation, and the presence of a family history of PNE were recorded. The severity of enuresis was classified as follows: mild if ≤2 episodes per week, moderate if 3–4 episodes per week, and severe if ≥5 episodes per week. A post-treatment enuresis frequency of less than two episodes per week was considered indicative of treatment success [8].

Serum levels of vitamin B12, 25-hydroxyvitamin D (25OHD), folate, and ferritin, as well as urinalysis, complete blood count, serum biochemistry, and urinary system ultrasonography findings routinely evaluated during follow-up visits based on physician recommendations, were recorded for all patients included in the study.

In children, serum 25OHD levels below 12 ng/mL were defined as vitamin D deficiency, while levels above 20 ng/mL were considered sufficient [13]. Serum vitamin B12 levels below 300 pg/mL were classified as low B12 levels [14,15]. Folate levels were interpreted as follows: <2 ng/mL as folate deficiency, 2–4 ng/mL as borderline folate insufficiency, and >4 ng/mL as normal [16]. Ferritin levels below 10 ng/mL were considered indicative of iron storage deficiency [17].

Patients were categorized based on their serum vitamin levels into those with deficiencies and those with normal values. Among the patients with vitamin deficiencies, three subgroups were established: Group 1 included patients with isolated vitamin B12 levels below the normal range; Group 2 included those with isolated 25-hydroxyvitamin D (25OHD) levels below the normal range; and Group 3 comprised patients with both vitamin B12 and 25OHD levels below the normal range. Formation of a control or placebo group was deemed unfeasible, since withholding treatment in patients with documented vitamin deficiencies and suboptimal (insufficient) levels would have raised significant ethical concerns.

All groups received a detailed reiteration of standard urotherapy protocols [18]. Patients in Groups 1, 2, and 3 were administered appropriate vitamin supplementation according to established clinical guidelines [19]. Each patient was instructed to maintain a voiding diary. During monthly follow-up visits, the number of wet nights per week was recorded.

The study was approved by the ethics committee of Fırat University on 3 July 2022 and was issued a protocol number on September 2025.

### Statistical Analysis

Statistical analyses were performed using IBM SPSS Statistics for Windows, Version 22.0 (Armonk, NY: IBM Corp.). Descriptive statistics were expressed as mean ± standard deviation for normally distributed continuous variables and as median (minimum–maximum) for non-normally distributed variables. Categorical variables were presented as counts and percentages (%).

## 3. Results

The study included 150 patients diagnosed with primary nocturnal enuresis (PNE). Patients enrolled in the study were between 5 and 17 years of age, with a calculated mean age of 8.78 ± 2.88 years. Of these, 85 (56.7%) were male and 65 (43.3%) were female, resulting in a male-to-female ratio of 1.3. The average number of wet nights per week was 6.39 ± 1.20. A history of recurrent urinary tract infections was reported in seven patients (4.7%).

All patients had normal blood pressure readings and no pathological findings were noted on physical examination. The mean body weight was 34.97 ± 19.81 kg (range: 15–108 kg), and the mean height was 134.17 ± 16.62 cm (range: 101–179 cm). No patients exhibited signs of delayed growth.

A positive family history of nocturnal enuresis was reported in 74 patients (49.3%), including 24 (16%) with a maternal history, 24 (16%) with a paternal history, and 26 (17.3%) with a history of nocturnal enuresis in other relatives (e.g., aunts, uncles, cousins). Regarding severity, enuresis was classified as mild in 2 patients (1.3%), moderate in 12 patients (8%), and severe in 136 patients (90.7%) (Table 1). In both patients presenting with mild enuresis, vitamin B12 deficiency was identified, and administration of vitamin B12 supplementation resulted in complete resolution of symptoms by the end of the three-month follow-up period.

Complete blood count, serum electrolytes, and blood urea and creatinine levels were within normal limits in all patients. No cases of persistent proteinuria or hematuria were detected on urinalysis. The mean urine specific gravity was 1021.5 (range: 1005–1030). Renal ultrasonography revealed grade 1 hydronephrosis in 7 patients (4.6%) and post-void residual urine in 17 patients (11.3%).

The mean serum vitamin B12 level was 242.51 ± 120.38 pg/mL (range: 63–1334 pg/mL), the mean serum 25-hydroxyvitamin D (25OHD) level was 22.04 ± 9.01 ng/mL (range: 4–58 ng/mL), the mean ferritin level was 22.56 ± 17.98 ng/mL (range: 3–145 ng/mL), and the mean folate level was 10.11 ± 3.65 ng/mL (range: 5–24.9 ng/mL).

Among the 150 patients diagnosed with PNE, only 21 (14%) had no detectable vitamin deficiencies. Low serum vitamin B12 levels were observed in 118 patients (78.6%), while decreased 25-hydroxyvitamin D (25OHD) levels were identified in 62 patients (41.3%). Concurrent below-normal levels of both 25OHD and vitamin B12 were observed in 51 patients (34%). Ferritin deficiency was detected in 26 patients (17.3%), all of whom also exhibited deficiencies in either vitamin B12 and/or 25OHD. No cases of folate deficiency or insufficiency were observed in the study cohort (Table 2).

**Figure 1:**
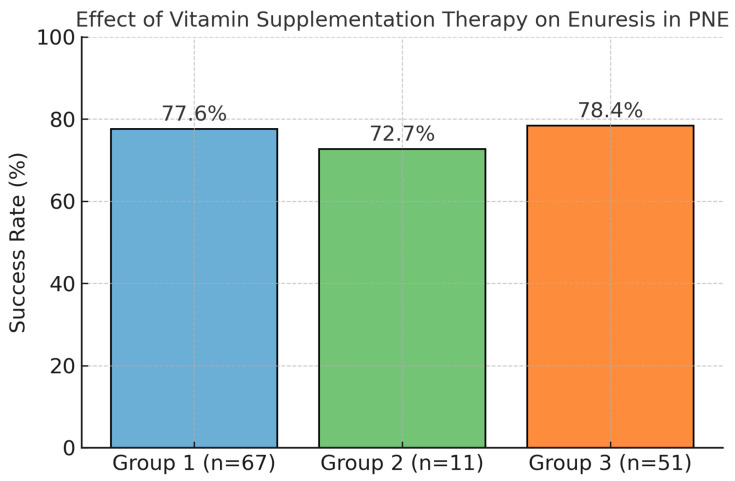
Children with primary nocturnal enuresis (PNE) and documented vitamin deficiencies were categorized into three groups. Following vitamin supplementation, the rates of improvement in enuresis were 77.6% in Group 1 (n = 67), 72.7% in Group 2 (n = 11), and 78.4% in Group 3 (n = 51), respectively.

Among 150 patients diagnosed with PNE, 129 (86%) were found to have vitamin levels below the normal range. Of these, 67 patients (44.66%) with isolated vitamin B12 deficiency/insufficiency were classified as Group 1; 11 patients (7.33%) with isolated 25-hydroxyvitamin D (25OHD) deficiency/insufficiency were classified as Group 2; and 51 patients (34%) with concurrent vitamin B12 and 25OHD deficiency/insufficiency were classified as Group 3 (Figure 1).

Patients in Groups 1, 2, and 3 received appropriate supplementation for their identified deficiencies/insufficiency. At the 3- to 6-month follow-up, the mean control values were as follows: vitamin B12, 356.7 ± 187.4 pg/mL; serum 25-hydroxyvitamin D (25OHD), 25.1 ± 7.0 ng/mL; and ferritin, 28.5 ± 8.8 ng/mL. After a follow-up period of approximately 3 to 6 months, treatment success, defined as enuresis occurring fewer than two nights per week, was achieved in 77.6%, 72.7%, and 78.4% of patients in Groups 1, 2, and 3, respectively. Of the patients in whom treatment success was achieved, 80% demonstrated a complete response. Despite adherence to recommendations, a total of 29 patients (22.4%) across the three groups continued to experience enuresis and were subsequently initiated on desmopressin therapy.

## 4. Discussion

In this study, we observed that 86% (n = 129) of the 150 children diagnosed with primary nocturnal enuresis (PNE) who had not responded to standard urotherapy had deficiencies in one or more of the following: vitamin B12, vitamin D, and ferritin. Notably, following supplementation of the deficient/insufficiency vitamins, 77.5% of these patients experienced a reduction in the frequency of enuresis to fewer than two nights per week. In our study, it was observed that a high rate of treatment success was achieved with this vitamin supplement. Accordingly, in patients with monosymptomatic enuresis, vitamin supplementation may represent an appropriate therapeutic option solely in the presence of documented deficiency or insufficiency.

PNE is known to negatively affect quality of life, leading to increased family stress, diminished academic performance, and impaired self-esteem. It can also contribute to emotional disturbances, such as anxiety, social isolation, and a decline in self-confidence [19,20,21]. Importantly, previous studies have demonstrated that improvements in self-confidence and social functioning can be observed following successful treatment of enuresis [22]. Therefore, early diagnosis and intervention are essential to mitigate these psychosocial consequences.

PNE is more common in boys than girls, particularly in younger age groups under 12 years of age [2]. In a previous study evaluating patients with monosymptomatic PNE, 57% of cases were reported to be male [8]. Similarly, in our study, 56.7% of the participants were boys, consistent with findings in the existing literature.

A recent study reported a family history of enuresis in 83.56% of parents of affected children [23]. In another study, the reported rates of parental enuresis were 7.1% for mothers and 7.1% for fathers [8]. In our cohort, 16% of patients had a maternal history of enuresis, and 16% had a paternal history, amounting to a combined parental history in 32% of cases. In the majority of patients who did not achieve treatment success despite vitamin supplementation (58.9%), a positive family history was noted. This observation raises the possibility that both genetic predisposition and environmental influences may substantially contribute to disease persistence.

In a recent population-based study including 3163 healthy children without chronic diseases, the mean serum vitamin B12 level was 374 pg/mL (range: 65–1747). B12 deficiency was reported in 5.5% of the children, borderline deficiency in 23.6%, and normal levels in 68.8% [24]. Another study involving 689 pediatric patients found that 23.2% had vitamin B12 deficiency and 49.3% had vitamin D deficiency during routine evaluations [25]. In contrast, our study in children with PNE revealed a markedly higher prevalence of vitamin deficiencies: 78.6% of patients had low serum B12 levels, and 41.3% had decreased 25OHD levels. Concurrent deficiencies in both B12 and 25OHD were observed in 34% of patients. These findings highlight a notably higher frequency of vitamin B12 deficiency in children with PNE compared to the general pediatric population.

A previous study in children with PNE reported vitamin D insufficiency in 48.3% of patients and vitamin D deficiency in 31.3%, along with a 25% prevalence of vitamin B12 deficiency. Additionally, the same study found that children who did not experience dry nights had significantly lower vitamin D levels [26]. In our study, however, vitamin B12 deficiency was more prominent, affecting 78.6% of patients diagnosed with PNE.

Recent research has highlighted the detrimental effects of nutritional deficiencies on cognitive and motor neuron functions in children [27]. Moreover, deficiencies in vitamin B12 and folate have been shown to contribute to delayed CNS maturation during adolescence, as well as behavioral alterations [27]. In a study examining B12, folate, and ferritin levels in children with PNE, vitamin B12 deficiency was found to be more prevalent in enuretic patients than in controls (40.7% vs. 25.6%; *p* = 0.037), while no cases of folate deficiency were reported in either group [15]. Our findings are consistent with these results and, given that both studies were conducted among pediatric populations in Turkey, we believe that regional dietary patterns may play a contributory role.

A study conducted in Egypt demonstrated that a group of 50 patients diagnosed with PNE had significantly lower serum 25-hydroxyvitamin D (25OHD) levels compared to healthy controls (19.0 ± 6.5 vs. 23.89 ± 4.19 ng/mL) [28]. In another study involving 30 children with PNE, both vitamin B12 and folate levels were significantly lower in the enuresis group compared to controls. Furthermore, the number of children with vitamin B12 deficiency was significantly higher in the PNE group than in the control group [5].

In the present study, all 26 patients with ferritin deficiency were concurrently found to have either vitamin B12 or vitamin D insufficiency, precluding the formation of a subgroup with isolated iron deficiency. Consequently, it remains unclear whether iron supplementation, when administered alongside other vitamin therapies, contributed independently to the achievement of dry nights.

There is a limited number of studies investigating the association between PNE and vitamin deficiencies, and the key findings from these studies are summarized in Table 3. Our findings are in agreement with several previous reports. However, in contrast to earlier studies, our investigation uniquely demonstrated a reduction in the number of wet nights following appropriate supplementation of the deficient/insufficient vitamins in children with PNE. Notably, while the beneficial effects of vitamin D supplementation on enuresis have been reported in only one study [29], the positive impacts of vitamin B12 supplementation on enuretic symptoms, as observed in our study, have not been previously documented in the literature.

A limitation of our study is the absence of a concurrently enrolled healthy control group without a diagnosis of PNE. However, data from the literature suggest that the prevalence and degree of vitamin B12 and 25OHD deficiencies among healthy children in our region are not as pronounced as those observed in our cohort of children with PNE [24,25].

In our study, only children who had previously received at least one month of standard urotherapy without symptomatic improvement were included, allowing us to evaluate the potential effect of vitamin supplementation. Nevertheless, it was not ethically feasible to form a comparative group of enuretic children with vitamin deficiencies who would receive repeated urotherapy without any vitamin supplementation, as withholding treatment in the presence of a documented deficiency would be inappropriate. Nevertheless, the lack of a control or placebo group constitutes the principal limitation of our study, precluding definitive conclusions as to whether the observed effects were truly independent of a potential placebo response.

## 5. Conclusions

We believe that our study will contribute both to the literature and to clinical practice, as it is the only study in the literature to document changes in nocturnal enuresis frequency in patients who were managed without desmopressin therapy, using only standard urotherapy combined with supplementation of specifically deficient/insufficient vitamin B12. Screening for vitamin B12 and D deficiencies, even in the absence of other symptoms, and the addition of a new supportive treatment to the management of PEN may lead to treatment success.

## Figures and Tables

**Table 1:** Demographic, clinical, and laboratory characteristics of the patients (n = 150).

Characteristic	Value
Age (years), mean ± SD (range)	8.78 ± 2.88
Sex, n (%)	Male: 85 (56.7) Female: 65 (43.3)
Male-to-female ratio	1.3
Number of wet nights per week, mean ± SD	6.39 ± 1.20
Body weight (kg), mean ± SD	34.97 ± 19.81
Height (cm), mean ± SD	134.17 ± 16.62
Family history, n (%)	74 (49.3) [Mother: 24 (16.0) Father: 24 (16.0) Others: 26 (17.3)]
Severity of enuresis, n (%)	Mild: 2 (1.3) Moderate: 12 (8.0) Severe: 136 (90.7)
Vitamin deficiencies, n (%)	None: 21 (14.0) Low B12: 118 (78.6) Low 25OHD: 62 (41.3) Low B12 + 25OHD: 51 (34.0)

**Table 2:** Serum levels of vitamin B12, 25-hydroxyvitamin D (25OHD), ferritin, and folate in patients with primary nocturnal enuresis.

Parameter	n (%)	Mean ± SD	Range
Vitamin B12 (pg/mL)		242.51 ± 120.38	63–1334
Normal (>300)	32 (21.4%)		
Low (<300)	118 (78.6%)		
Deficient (<200)	66 (44.0%)		
25-Hydroxyvitamin D (ng/mL)		22.04 ± 9.01	4–58
Normal (>20)	88 (58.7%)		
Low (<20, total)	62 (41.3%)		
Deficient (<12)	19 (12.6%)		
Ferritin (ng/mL)		22.56 ± 17.98	3–145
Normal (≥10)	124 (82.7%)		
Low (<10)	26 (17.3%)		
Folate (ng/mL)		10.11 ± 3.65	5–24.9
Normal (>4)	150 (100.0%)		
Low (2–4)	0 (0.0%)		
Deficient (<2)	0 (0.0%)		

**Table 3:** Studies investigating vitamin B12, 25-hydroxyvitamin D (25OHD), and folate levels in patients diagnosed with primary nocturnal enuresis.

Authors	Year	Objective	Study Design	Sample Size (Patient/Control)	Key Findings
Altunoluk et al. [5]	2012	To evaluate the relationship between vitamin B12/folate levels and neurogenic maturation in children with PEN	Cross-sectional study	30/31	Significantly lower B12 and folate levels in PEN group
Siroosbakht et al. [8]	2023	To investigate the association between vitamin D levels and the development/severity of PEN	6-year case–control study	267/267	Vitamin D levels significantly lower in PEN group
Zeybek et al. [15]	2024	To assess serum B12, folate, and ferritin levels in children with PEN	Retrospective, single-center	86/90	B12 and folate levels significantly lower; no difference in ferritin
Keleş et al. [23]	2024	To evaluate vitamin levels in PEN	Retrospective cohort study	146/102	Significantly lower B12, folate, and vitamin D levels in PEN group
Ibrahim et al. [26]	2024	To assess the relationship between vitamin D/B12 deficiency and PEN severity	Cross-sectional study	288/—	Vitamin D deficiency common and associated with PEN severity
El-Baz et al. [28]	2021	To compare vitamin D levels in children with and without PEN	Case–control study	50/50	Vitamin D levels significantly lower in PEN group
Rahmani et al. [29]	2018	To assess the effect of vitamin D and omega-3 on enuresis	Observational study	162/—	Vitamin D found beneficial for PEN
Kompani et al. [30]	2023	To evaluate serum B12 and folic acid levels in children with PEN	Case–control study	43/99	Significantly lower B12 and folic acid levels in PEN group
Li et al. [31]	2014	To examine the association between 25OHD levels and enuresis	Observational study	43 PEN cases/247 total	Higher prevalence of PEN among children with low vitamin D
Albayrak et al. [32]	2015	To compare levels of B12, folate, and iron in PEN	Comparative study	40/30	B12 and folate lower; iron levels higher in PEN group
Mostafa et al. [33]	2024	To evaluate the association between vitamin D deficiency and PEN	Case–control study	60/60	Significant difference in vitamin D status between groups

## Data Availability

Datasets generated or analyzed in this study are accessible upon reasonable request from the corresponding author.
